# Giant proliferating trichilemmal cyst

**DOI:** 10.11604/pamj.2014.18.195.4354

**Published:** 2014-07-05

**Authors:** Mariem Mohamed, Yosra Soua

**Affiliations:** 1 Dermatology Department, Monastir University Hospital, Monastir, Tunisia

**Keywords:** Trichilemmal cyst, diabetes, painless mass, ulcer

## Image in medicine

**Figure 1:**
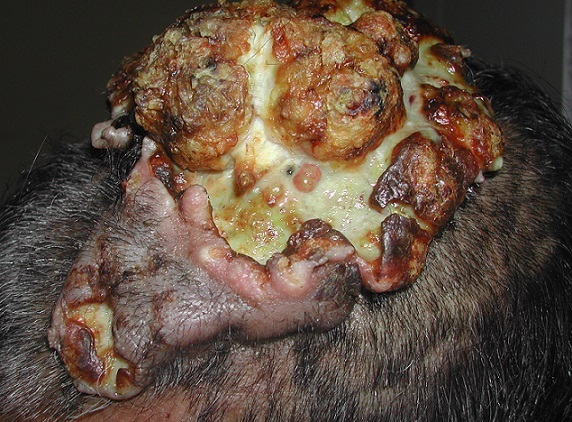
The mass was solid in consistency, mobile, measuring about 10 cm x 5 cm x 3 cm

A 78-year-old man with diabetes under insulin was referred to our department for a 30-year history of lobulated, painless mass on the frontal scalp area. It was initially growing slowly and later it started to ulcerate with foul smelling discharge and rapid enlargement that interfered with the patient's daily life mainly during the last 6 months. A physical examination showed a huge tumoral lesion, oval in shape, situated in the central frontal area slightly to the left side with an irregular surface area. It was associated with ulceration as well as serous discharge but with no bleeding. The mass was solid in consistency, mobile, measuring about 10 cm×5 cm ×3 cm (Figure 1). The surrounding skin was intact. Further examination revealed no palpable lymph nodes. We performed complete excision of the lesion. Histological examination showed cyst cavity with characteristic amorphous eosinophilic keratin regions of trichilemmal keratinization, variable cytologic atypia and mitotic activity together with lobulation of the cyst wall and pilling up of the squamous epithelium. The stroma exhibited numerous vessels and granulation tissue on epidermal hyperplasia. The correlation of clinical findings with histopathology confirmed the diagnosis of proliferating trichilemmal cyst.

